# Posterior teeth angulation in non-extraction and extraction treatment of anterior open-bite patients

**DOI:** 10.1186/s40510-017-0167-z

**Published:** 2017-06-05

**Authors:** Guilherme Janson, Mayara Rizzo, Vinicius Laranjeira, Daniela Gamba Garib, Fabricio Pinelli Valarelli

**Affiliations:** 1 Department of Orthodontics, Bauru Dental School, University of São Paulo, Alameda Octávio Pinheiro Brisolla 9-75, Bauru, SP 17012-901 Brazil; 2 Department of Orthodontics, Ingá Dental School, Maringá, PR Brazil

## Abstract

**Backgound:**

This study cephalometrically evaluated the posterior teeth angulation changes of anterior open-bite non-extraction and extraction treatment in the permanent dentition, with anterior vertical elastics.

**Methods:**

The sample consisted of initial and final lateral headfilms of 60 patients divided into 2 groups: Group 1 consisted of 30 patients treated with non-extraction with an initial mean age of 15.26 years and treated with fixed appliances for a mean period of 2.46 years. Group 2 consisted of 30 patients treated with extractions, with an initial mean age of 14.03 years, and treated with fixed appliances for a mean period of 2.49 years. Within-group treatment changes were evaluated with paired *t* tests. Results were considered statistically significant at *P* < 0.05.

**Results:**

The mandibular posterior teeth were significantly uprighted in both groups with both treatment protocols.

**Conclusions:**

Correction of anterior open bite with either non-extraction or extractions with continuous archwires and vertical anterior elastics uprights the mandibular posterior teeth.

## Background

Open-bite treatment should ideally be performed during the deciduous or mixed dentition, when stability of the results is very high [1–3]. When performed during the permanent dentition, treated whether with non-extraction, with extractions, or even orthodontic surgically, there are varying degrees of clinically significant relapse [4–8].

Generally, open-bite malocclusions have some skeletal involvement, presenting divergence of the palatal and mandibular planes [9–13]. Regarding the dentoalveolar characteristics, Kim [14] suggested that the posterior teeth are more mesially angulated in open-bite patients and developed the multiloop edgewise archwire technique, which incorporates distal tip-back bends to the posterior teeth in order to upright them. Anterior vertical intermaxillary elastics are also used to provide the vertical forces to close the anterior open bite [14].

An alternative method to upright the posterior teeth, instead of incorporating tip-back bends on the archwires, is to mesially angulate the posterior teeth accessories and use continuous archwires associated with anterior vertical intermaxillary elastics [14–17]. This procedure may simplify the orthodontic mechanics and provide more patient comfort. It is also speculated that the procedure of uprighting the posterior teeth will provide more treatment stability [14, 17].

Although the principle of intentionally uprighting the posterior either with tip-back bends on the archwires or by mesially angulating the posterior teeth accessories has been developed some time ago, this principle was not generally used [14, 17]. The most common procedure to close anterior open bite, before mini-implant [18–20] and mini-plate [21–23] development, was to use anterior vertical intermaxillary elastics, especially in non-extraction protocols [14–17]. However, it is quite obvious that some uprighting of the posterior teeth may occur even if no tip-back bends are incorporated or the accessories of the posterior teeth are not mesially tipped, due to the action of the elastic forces on these teeth [15–17, 24].

Therefore, the objective of this study is to investigate whether the posterior teeth are able to be uprighted even when no tip-back bends are incorporated or the accessories of the posterior teeth are not mesially tipped, in non-extraction and extraction open-bite treatments.

## Methods

This study was approved by the Ethics in Research Committee of Bauru Dental School, University of São Paulo, Brazil.

**Fig. 1:**
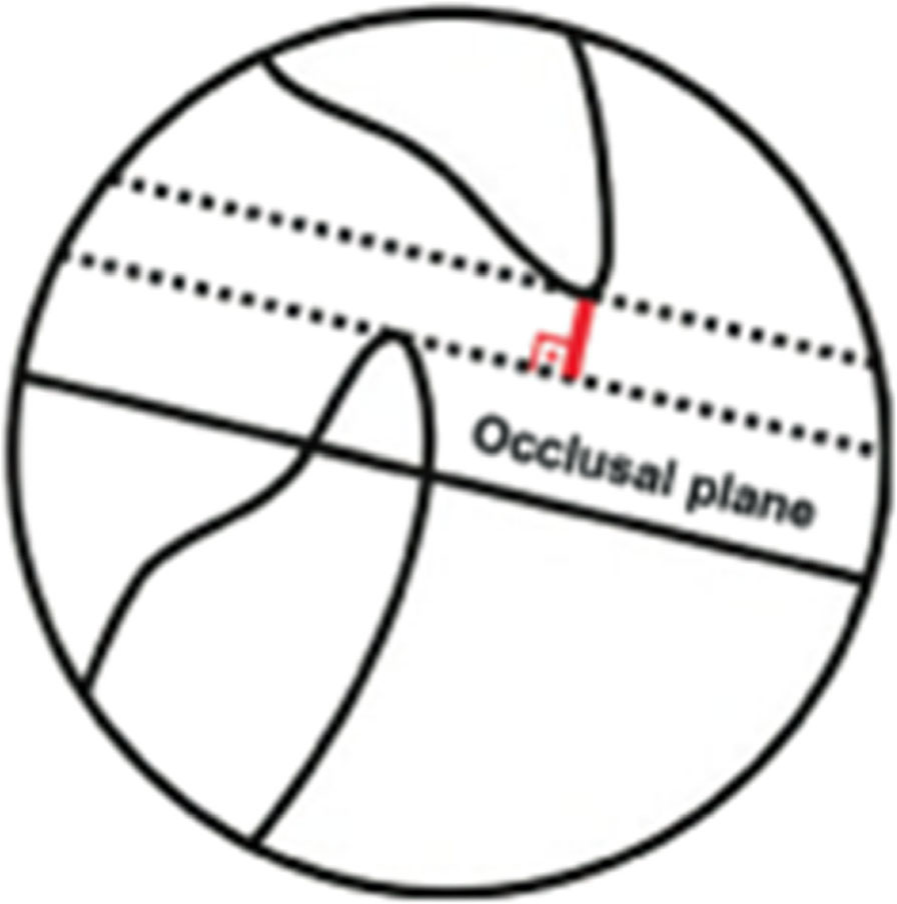
Overbite measurement was defined as the distance between the maxillary and mandibular incisor borders perpendicularly to the functional occlusal plane

The study comprised 60 open-bite treated patients of both sexes, selected from the files of the Orthodontic Department at Bauru Dental School, University of São Paulo, divided into two groups: Group 1 consisted of 30 (12 male; 18 female) patients treated with non-extraction, with mean pre- and posttreatment ages of 15.26 (SD = 3.99) and 17.73 (SD = 4.16) years, respectively, and an initial mean open bite of 1.47 mm. Ten patients presented with Class I, 18 with Class II, and 2 with Class III malocclusions. Ten patients underwent maxillary expansion either with hyrax or Haas appliances to correct posterior crossbites or to provide space in the maxillary arch [25, 26]. Group 2 included 30 patients (11 male; 19 female), treated with extractions, with mean pre- and posttreatment ages of 14.03 (SD = 2.70) and 16.53 (SD = 3.12) years, respectively, with an initial mean open bite of 1.93 mm. Fourteen patients presented with Class I, 15 with Class II, and 1 with Class III malocclusions. Twenty-seven were treated with four first premolar extractions, 2 were treated with two first maxillary premolar extractions, and 1 was treated with two first mandibular premolar extractions. Seven underwent maxillary expansion either with hyrax or Haas appliances to correct posterior crossbites or to provide space in the maxillary arch. The main selection criterion was that the patients should present initially an anterior open bite clinically greater than or equal to 1 mm (Fig. 1). Additional criteria were the presence of all maxillary and mandibular teeth up to the first molars and that the patients had been treated non-extraction and with extractions with Edgewise appliances.

In the non-extraction group, treatment was conducted with the standard Edgewise technique, which is characterized by the use of 0.022 × 0.028-in. conventional brackets. For leveling and alignment, the usual wire sequence begins with a 0.015-in. twist-flex or a 0.016-in. nickel-titanium wire, followed by 0.016, 0.018, and 0.020-in. stainless steel round wires. Vertical anterior elastics to correct the open bite usually began to be used when at least an 0.018-in. stainless steel round archwire was inserted. The elastics were used until a positive overbite was obtained. Detailing of tooth position and the finishing procedures were accomplished by either 0.019 × 0.025-in. or 0.021 × 0.025-in. rectangular wires and 0.018-in. round wires, respectively. No additional auxiliaries were used to control the vertical dimension. After the active treatment period, a Hawley retainer was used in the maxillary arch and a bonded canine-to-canine retainer in the mandibular arch. Myofunctional therapy was recommended to correct tongue posture and function, when necessary. The mean treatment time was 2.46 years.

In the extraction group, the appliances used were similar to the non-extraction group. After the extractions, if there were anterior crowding, the canines were initially retracted until the anterior teeth could be aligned. Thereafter, leveling and alignment progressed as in the non-extraction group until a rectangular wire was inserted. Vertical anterior intermaxillary elastics were usually used after insertion of a 0.018-in. round stainless steel archwire. The elastics were used until a positive overbite was obtained. Maxillary and mandibular anterior retraction were performed on the rectangular archwires with elastic chains. Anchorage reinforcement was provided with an extraoral headgear in the maxillary and a lip bumper in the mandibular arch, when necessary. The retention procedures were similar to the non-extraction group. Myofunctional therapy was recommended to correct tongue posture and function, when necessary. The mean treatment time was also 2.49 years.

**Fig. 2:**
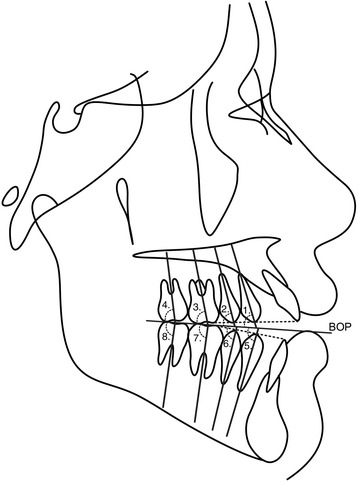
Posterior teeth angulations measurements in the maxillary and in the mandibular arch related to bisected occlusal plane (*BOP*). (1) Mx4.BOP (°)—long axis (apex to tip) of maxillary 1st premolar to bisected occlusal plane (BOP). (2) Mx5.BOP (°)—long axis (apex to tip) of maxillary 2nd premolar to bisected occlusal plane (BOP). (3) Mx6.BOP (°)—long axis (furcation to center of the crown) of maxillary 1st molar to bisected occlusal plane (BOP). (4) Mx7.BOP (°)—long axis (furcation to center of the crown) of maxillary 2nd molar to bisected occlusal plane (BOP). (5) Md4.BOP (°)—long axis (apex to tip) of mandibular 1st premolar to bisected occlusal plane (BOP). (6) Md5.BOP(°)—long axis (apex to tip) of mandibular 2nd premolar to bisected occlusal plane (BOP). (7) Md6.BOP (°)—long axis (furcation to center of the crown) of mandibular 1st molar to bisected occlusal plane (BOP). (8) Md7.BOP (°)—long axis (furcation to center of the crown) of mandibular 2nd molar to bisected occlusal plane (BOP)

**Fig. 3:**
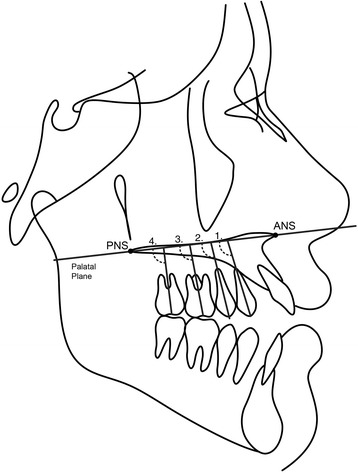
Posterior teeth angulations measurements in the maxillary arch related to palatal plane (PP). (1) Mx4.PP (°)—long axis (apex to tip) of maxillary 1st premolar to palatal plane (ANS–PNS); (2) Mx5.PP (°)—long axis (apex to tip) of maxillary 2nd premolar to palatal plane (ANS–PNS); (3) Mx6.PP (°)—long axis (furcation to center of the crown) of maxillary 1st molar to palatal plane (ANS–PNS); (4) Mx7.PP (°)—long axis (furcation to center of the crown) of maxillary 2nd molar to palatal plane (ANS–PNS)

**Fig. 4:**
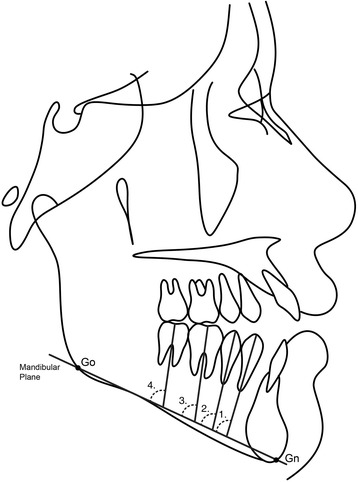
Posterior teeth angulations measurements in the mandibular arch related to mandibular plane (M.P). (1) Md4.MP (°)—long axis (apex to tip) of mandibular 1st premolar to mandibular plane (Go–Gn); (2) Md5.MP (°)—long axis (apex to tip) of mandibular 2nd premolar to mandibular plane (Go–Gn); (3) Md6.MP (°)—long axis (furcation to center of the crown) of mandibular 1st molar to mandibular plane (Go–GN); (4) Md7.MP (°)—long axis (furcation to center of the crown) of mandibular 2nd molar to mandibular plane (Go–Gn)

**Fig. 5:**
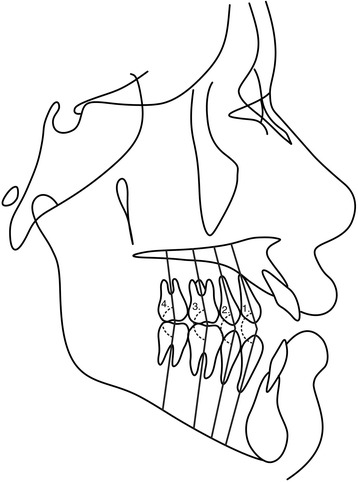
Interpremolar and intermolar angulations measurements. (1) Mx4.Md4 (°)—long axis (apex to tip) of maxillary and mandibular 1st premolars; (2) Mx5.Md5 (°)—long axis (apex to tip) of maxillary and mandibular 2nd premolars; (3) Mx6.Md6 (°)—long axis (furcation to center of the crown) of maxillary and mandibular 1st molars; (4) Mx7.Md7 (°)—long axis (furcation to center of the crown) of maxillary and mandibular 2nd molars

Two lateral cephalometric headfilms were obtained from each patient at the pre- (T1) and posttreatment (T2) stages. The cephalometric tracings and landmark identifications were performed on acetate paper by a single investigator (M.R.) and then digitized with a DT-11 digitizer (Houston Instruments, Austin, TX). In total, 128 landmarks were identified and 30 measurements were performed (Table 1 and Figs. 2, 3, 4, and 5). These data were stored in a computer and analyzed with Dentofacial Planner 7.0 (Dentofacial Planner Software, Toronto, Ontario, Canada), which corrected the image magnification factors of the groups. The magnification factors varied from 6 to 10.94%, because the radiographs were taken on different X-ray machines.

**Table 1:** Cephalometric variables

Maxillomandibular relationship	
SNA (°)	SN to NA angle
SNB (°)	SN to NB angle
ANB (°)	NA to NB angle
Growth pattern	
SN.PP (°)	SN to palatal plane angle
SN.GoGn (°)	SN to Go–Gn angle
PP.MP (°)	Angle between palatal plane(ANS/PNS) and mandibular plane(Go/Gn)
FMA (°)	Frankfurt horizontal to mandibular plane angle
Gonial angle (°)	Ar–Go to Go–Gn angle
LAFH (mm)	Lower anterior face height: distance from ANS to menton
Dental relationship	
Overbite (mm)	Distance between the maxillary and mandibular incisor borders.
Maxillary teeth mesio-distal angulations	
Mx4.BOP (°)	Long axis (apex to tip) of maxillary 1st premolar to bisected occlusal plane (BOP)
Mx5.BOP (°)	Long axis (apex to tip) of maxillary 2nd premolar to bisected occlusal plane (BOP)
Mx6.BOP (°)	Long axis (furcation to center of the crown) of maxillary 1st molar to bisected occlusal plane (BOP)
Mx7.BOP(°)	Long axis (furcation to center of the crown) of maxillary 2nd molar to bisected occlusal plane (BOP)
Mx4.PP (°)	Long axis (apex to tip) of maxillary 1st premolar to palatal plane(ANS–PNS)
Mx5.PP (°)	Long axis (apex to tip) of maxillary 2nd premolar to palatal plane (ANS–PNS)
Mx6.PP (°)	Long axis (furcation to center of the crown) of maxillary 1st molar to palatal plane (ANS–PNS)
Mx7.PP (°)	Long axis (furcation to center of the crown) of maxillary 2nd molar to palatal plane (ANS–PNS)
Mandibular teeth mesio-distal angulations	
Md4.BOP (°)	Long axis (apex to tip) of mandibular 1st premolar to bisected occlusal plane (BOP)
Md5.BOP (°)	Long axis (apex to tip) of mandibular 2nd premolar to bisected occlusal plane (BOP)
Md6.BOP (°)	Long axis (furcation to center of the crown) of mandibular 1st molar to bisected occlusal plane (BOP)
Md7.BOP (°)	Long axis (furcation to center of the crown) of mandibular 2nd molar to bisected occlusal plane(BOP)
Md4.MP (°)	Long axis (apex to tip) of mandibular 1st premolar to mandibular plane (Go–Gn)
Md5.MP (°)	Long axis (apex to tip) of mandibular 2nd premolar to mandibular plane (Go–Gn)
Md6.MP (°)	Long axis (furcation to center of the crown) of mandibular 1st molar to mandibular plane (Go–Gn)
Md7.MP (°)	Long axis (furcation to center of the crown) of mandibular 2nd molar to mandibular plane (Go–Gn)
Interdental angulations	
Mx4.Md4 (°)	Long axis (apex to tip) of maxillary and mandibular 1st premolars
Mx5.Md5 (°)	Long axis (apex to tip) of maxillary and mandibular 2nd premolars
Mx6.Md6 (°)	Long axis (furcation to center of the crown) of maxillary and mandibular 1st molars
Mx7.Md7 (°)	Long axis (furcation to center of the crown) of maxillary and mandibular 2nd molars

## Error study

Thirty randomly selected radiographs were retraced, redigitized, and remeasured by the same examiner (M.R.) after a 30-day interval. The random errors were calculated according to Dahlberg’s formula (Se^2^ = Σ*d*2/2*n*) [27], where Se^2^ is the error variance and *d* is the difference between two determinations of the same variable and the systematic errors with dependent *t* tests, at *P* < 0.05 [28–30].

## Statistical analyses

Normal distribution of the variables was evaluated with Kolmogorov–Smirnov tests. Results of this test demonstrated that all variables were normally distributed. Therefore, paired *t* tests were used for intra-group comparison to evaluate the treatment changes within each group.

Results were regarded as significant at *P* < 0.05. These analyses were performed with Statistica software (Statistica for Windows 10.0; Statsoft, Tulsa, OK).

## Results

No significant systematic errors were detected, and the random errors varied between 0.16 (OB) and 4.29 (Mx7.Md7) degrees.

In the non-extraction group, the maxillary second molar was significantly mesially angulated in relation to the BOP. The mandibular premolars and molars were significantly distally angulated with treatment, regarding BOP and the mandibular plane. There were significant increases in the interfirst and intersecond premolars and interfirst molar angles (Table 2).

**Table 2:** Comparison of cephalometric variables between pretreatment and posttreatment stages for the non-extraction group (dependent *t* tests)

Variables (°)	Initial T1		Final T2		*P*
	Mean	SD	Mean	SD	
Maxillary teeth mesio-distal angulations					
Mx4.BOP(*n* = 30)	79.26	6.15	79.74	5.71	0.679
Mx5.BOP(*n* = 30)	85.64	5.79	86.76	5.92	0.341
Mx6.BOP(*n* = 30)	91.71	6.88	89.06	4.99	0.103
Mx7.BOP(*n* = 27)	101.52	8.10	97.53	6.51	0.018*
Mx4PP (*n* = 29)	90.78	6.22	89.38	7.19	0.215
Mx5PP (*n* = 29)	84.40	6.22	82.37	8.59	0.092
Mx6PP (*n* = 30)	78.33	7.43	80.07	5.42	0.268
Mx7PP (*n* = 27)	68.77	8.70	71.91	8.37	0.070
Mandibular teeth mesio-distal angulations					
Md4.BOP(*n* = 30)	77.19	5.51	81.30	4.74	0.000*
Md5.BOP(*n* = 30)	77.26	5.12	83.16	5.04	0.000*
Md6.BOP(*n* = 30)	75.26	5.63	83.80	5.78	0.000*
Md7.BOP(*n* = 27)	69.52	5.70	77.68	7.87	0.000*
Md4MP (*n* = 30)	79.06	5.52	75.48	5.22	0.000*
Md5MP (*n* = 30)	78.98	5.03	73.61	4.94	0.000*
Md6MP (*n* = 30)	80.98	7.06	72.96	5.69	0.000*
Md7MP (*n* = 27)	86.72	7.23	79.16	6.46	0.000*
Interdental angulations					
Mx4.Md4 (*n* = 29)	156.46	8.88	161.05	6.42	0.003*
Mx5.Md5 (*n* = 29)	162.90	7.83	169.21	6.74	0.000*
Mx6.Md6 (*n* = 30)	166.85	8.07	171.29	5.45	0.023*
Mx7.Md7 (*n* = 27)	171.79	9.03	175.49	9.59	0.060

*Statistically significant at *P* < 0.05

In the extraction group, all maxillary posterior teeth were significantly mesially angulated and the mandibular posterior teeth were significantly distally angulated. Only the maxillary second premolar was not significantly mesially angulated regarding the BOP. There was a significant increase in the intersecond premolar and a significant decrease in the intersecond molar angle (Table 3).

**Table 3:** Comparison of cephalometric variables between pretreatment and posttreatment stages for the extraction group (dependent *t* tests)

Variables (°)	Initial T1		Final T2		*P*
	Mean	SD	Mean	SD	
Maxillary teeth mesio-distal angulations					
Mx5.BOP(*n* = 30)	85.26	3.88	83.64	4.43	0.156
Mx6.BOP(*n* = 30)	89.94	4.14	87.00	4.91	0.031*
Mx7.BOP(*n* = 24)	102.32	5.24	94.00	5.85	0.000*
Mx5PP (*n* = 30)	82.17	6.31	85.02	6.37	0.026*
Mx6PP (*n* = 30)	77.49	6.56	81.66	6.88	0.007*
Mx7PP (*n* = 24)	65.70	6.88	74.76	6.33	0.000*
Mandibular teeth mesio-distal angulations					
Md5.BOP(*n* = 30)	77.44	5.99	82.19	5.01	0.001*
Md6.BOP(*n* = 30)	76.92	6.10	82.21	5.61	0.000*
Md7.BOP(*n* = 24)	73.54	5.24	78.37	6.47	0.003*
Md5MP (*n* = 30)	78.60	6.20	72.85	6.88	0.000*
Md6MP (*n* = 30)	78.49	6.85	72.83	6.77	0.000*
Md7MP (*n* = 24)	80.85	5.70	76.39	7.50	0.001*
Interdental angulations					
Mx5.Md5 (*n* = 30)	162.00	6.42	165.67	7.91	0.045*
Mx6.Md6 (*n* = 30)	166.78	6.91	168.62	6.02	0.150
Mx7.Md7 (*n* = 24)	175.45	9.65	168.57	7.09	0.002*

*Statistically significant at *P* < 0.05

The power of the tests was based on the results of intragroup comparisons of the current investigation. With a standard deviation of 7.94 for the Md6.BOP variable, and a minimum difference of 4.2° to be detected, with 30 subjects in the group, the test power was of 80%.

## Discussion

### Sample selection and study design

The specific criterion for sample selection in this study was that the patients presented an open bite of at least 1 mm, which is similar to other investigations [6–8, 13, 31]. No effort was made to include only open bites with predominant skeletal features. Therefore, the mean characteristics of the groups represent the predominant type of patient in an orthodontic practice, and the results are mostly adequate for these patients.

The random errors ranged from 0.16 (over-bite) to 4.29 (Mx7.Md7). The random errors do not significantly compromise the validity of this work, since four landmarks are needed to obtain these angles and some of these landmarks are subjective [32–34]. Therefore, a few random errors of greater magnitude were expected. Similar studies do not mention the random error magnitude [16, 24]. Therefore, probably, they would be similar for the mentioned reasons.

The decision to use non-extraction and extraction patients was because there are some differences in treatment mechanics between these groups [14–16, 24, 31]. Basically, when treating non-extraction, the open bite is closed only consequent to the anterior vertical intermaxillary elastics [13, 14, 16, 35, 36]. In the extraction group, besides the elastics, the open bite is also closed through the drawbridge principle and by some mesialization of the posterior teeth [35, 37]. Therefore, there was a need to know whether these procedures would influence the angulation of the posterior teeth.

### Posterior teeth angulation

In the non-extraction group, the treatment changes caused a significant mesial angulation of the maxillary second molar in relation to BOP (Table 2). This is contrary to what would be expected [13, 14]. The explanation may be based on its eruption orientation. Before eruption, the second molar crown is usually distally angulated, and after eruption, as the teeth occlude, the apex moves distally, uprighting it [38, 39]. The patients began treatment at a mean age of 15.26 years, in which the second molars are still more distally angulated than their final position [38, 39]. As the teeth are leveled and aligned, it was uprighted by these procedures [15, 16, 24].

In the mandibular arch, all posterior teeth were significantly distally angulated with treatment (Table 2). This shows that even without intentionally trying to distally tip these teeth with tip-back bends in the archwires or by mesially angulating the posterior teeth accessories, it is possible to upright, at least, the mandibular posterior teeth. If uprighting the posterior teeth actually leads to more stability, as speculated [13, 14, 16, 17, 24], this may have contributed to it in an earlier study with some of these patients [6]. Therefore, the principle of uprighting the posterior teeth can be partially accomplished even without the mentioned procedures [13–16, 24]. This uprighting may also be seen as a necessary dentoalveolar change to compensate for the divergent palatal and mandibular planes that are usually associated with open-bite characteristics [40].

The mandibular premolars distal tipping significantly increased the interpremolar and interfirst molar angles (Table 2). However, the mandibular second molars distal tipping was not enough to significantly increase the intersecond molar angles.

In the extraction group, the treatment changes caused significant mesial angulation of all the maxillary teeth (Table 3). This is most likely due to the extraction mechanics that may have allowed some mesialization of these teeth, through mesial tipping [41]. Additionally, for the second molars, the eruption orientation may have also contributed to a certain amount, as explained for the non-extraction group.

In the mandibular arch, all posterior teeth were significantly distally angulated with treatment (Table 3). The same explanation given to the non-extraction group applies here. Additionally, it has to be emphasized that despite some tendency that the posterior teeth have to mesially tip during space closure [41], the mechanics with vertical elastics was able to overcome it and distally tip them.

The mandibular second premolar and molar distal tipping was able to overcome the maxillary second premolar and molar mesial tipping and significantly increased the intersecond premolar and intersecond molar angles, respectively (Table 3). However, the mandibular first molars distal tipping was not enough to overcome the maxillary first molars mesial tipping and significantly increase the intermolar angles.

### Clinical implications

This study showed that posterior teeth uprighting is usually obtained when using continues archwires and anterior vertical elastics to close anterior open bite, especially in the mandibular arch. However, uprighting of the posterior maxillary teeth is more difficult, primarily in extraction cases. Therefore, mechanical procedures such as the multiloop edgewise archwire technique [13, 14] or mesially angulating the posterior teeth accessories with continuous archwires [15–17, 24] are recommended to increase the possibility of actually uprighting the posterior teeth. If, in fact, this mesial angulation increases treatment stability, this will be a contributing factor. Nevertheless, despite some clinical evidence that uprighting the posterior teeth will increase treatment stability, this has yet to be investigated.

## Conclusions

The conclusions are the following:

- Correction of anterior open bite with either non-extraction or extractions with continuous archwires and vertical anterior elastics uprights the mandibular posterior teeth.
- However, there is also mesial angulation of the maxillary posterior teeth with this mechanics.
- Therefore, additional mechanical procedures should be used to increase posterior teeth uprighting in open-bite treatment, especially in the maxillary arch.
